# An Unusual Presentation of Carney Complex

**DOI:** 10.4274/jcrpe.galenos.2019.2019.0043

**Published:** 2020-03-19

**Authors:** Aydilek Dağdeviren Çakır, Hande Turan, Tiraje Celkan, Nil Çomunoğlu, Oya Ercan, Olcay Evliyaoğlu

**Affiliations:** 1 İstanbul University-Cerrahpaşa, Cerrahpaşa Faculty of Medicine, Department of Pediatric Endocrinology, İstanbul, Turkey; 2 İstanbul University-Cerrahpaşa, Cerrahpaşa Faculty of Medicine, Department of Pediatric Hematology-Oncology, İstanbul, Turkey; 3 İstanbul University-Cerrahpaşa, Cerrahpaşa Faculty of Medicine, Department of Pathology, İstanbul, Turkey

**Keywords:** Carney complex, osteochondromyxoma, large cell calcifying Sertoli cell tumor, central puberty precocious

## Abstract

Carney complex (CNC) is a multiple neoplasia syndrome, characterized by pigmented lesions of the skin and mucosa, cardiac, cutaneous and other myxomas and multiple endocrine and non-endocrine tumors. Most of the cases have an inactivating mutation in the *PRKAR1A* gene. Osteochondromyxoma (OMX) is an extremely rare myxomatous tumor of bone, affecting 1% of CNC patients. Large cell calcifying Sertoli cell tumor (LCCSCT) is a testicular tumor affecting more than 75% of males with CNC. Here, we report an atypical case of CNC without typical pigmented skin lesions, presenting with a bone based tumor as the first manifestation. Initial presentation was for a recurrent, locally invasive intranasal tumor without definite diagnosis. Further clinical developments during follow up, central precocious puberty and testicular tumor with calcification, led to the diagnosis of LCCSCT, a CNC-related tumor. Histopathologic examination of the intranasal tumor was re-evaluated with this knowledge and OMX was diagnosed. Coexistence of OMX and LCCSCT suggested CNC. Genetic analysis revealed a heterozygous non-sense p.Trp 224* (c.672G>A) in the PRKAR1A gene. In our case, the diagnosis of OMX was delayed, because it is extremely rare and little is known about this tumor. Thus the aim of this report was to alert other clinicians to consider CNC if OMX is diagnosed.

**What is already known on this topic?**

Carney complex (CNC) is a multiple neoplasia syndrome characterized by pigmented lesions of the skin and mucosa, cardiac, cutaneous and other myxomas, and multiple endocrine and non-endocrine tumors. Osteochondromyxoma (OMX) is an extremely rare myxomatous tumor of bone, affecting 1% of CNC patients.

**What this study adds?**

CNC may present without typical findings such as pigmented skin lesions. This case presented initially with OMX although this diagnosis was delayed. The patient was subsequently diagnosed with large cell calcifying Sertoli cell tumor, leading to the final diagnosis of CNC. Clinicians should consider CNC if OMX is diagnosed.

## Introduction

Carney complex (CNC) is a rare multiple neoplasia syndrome with an autosomal dominant inheritance (1). However, approximately 30% of cases occur sporadically, as a result of *de novo* mutations (2). Most patients with CNC, have inactivating mutations in the protein kinase CAMP-dependent type-1 regulatory subunit alpha (*PRKAR1A*) gene. *PRKAR1A* may act as a tumor suppressor gene by regulating protein kinase A activity, which in turn can suppress or stimulate cell growth and differentiation (3).

CNC is characterized with spotty pigmentation of the skin, endocrinopathy and endocrine and nonendocrine tumors. Patients often have tumors of two or more endocrine glands, including adrenal cortex, the pituitary, thyroid, and gonads. This syndrome is associated with many other non-endocrine tumors, including cardiac myxomas, psammomatous melanotic schwannomas, breast myxomas, osteochondromyxomas (OMX) and abnormal pigmentation (lentiginious) or myxomas of the skin (2,4).

Here we report an atypical case of CNC. He had initially presented with an intranasal tumor, although the pathologic diagnosis was not clearly established. During follow-up, large cell calcifying Sertoli cell tumor (LCCSCT), a CNC related tumor, was detected in the testes and with this knowledge, the intranasal tumor was re-evaluated histopathologically and was found to be an OMX, a rare component of CNC. Coexistence of OMX and LCCSCT suggested CNC and a previously reported mutation, c.672G>A (p.Trp224*) was detected heterozygously in the *PRKAR1A* gene.

## Case Report

A 9-year-old male was referred to our pediatric endocrinology department because of partial empty sella on pituitary magnetic resonance imaging (MRI), detected during follow up of an intranasal tumor.

Patient clinical history revealed that he had been admitted to another hospital three years previously with the complaint of swelling around his right orbita. Orbital MRI had revealed an intranasal tumor filling the nasal sinuses. The tumor was invading and degrading the cribriform plate and orbita medial wall. This tumor was excised and osteochondroid tissue with osteoblasts, which suggested fibrous dysplasia histopathologically, was identified.

**Figure 1:**
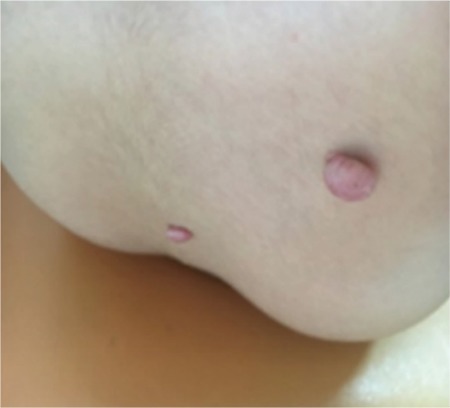
Cutaneous myxomas, the largest with a diameter of 5 mm

**Figure 2:**
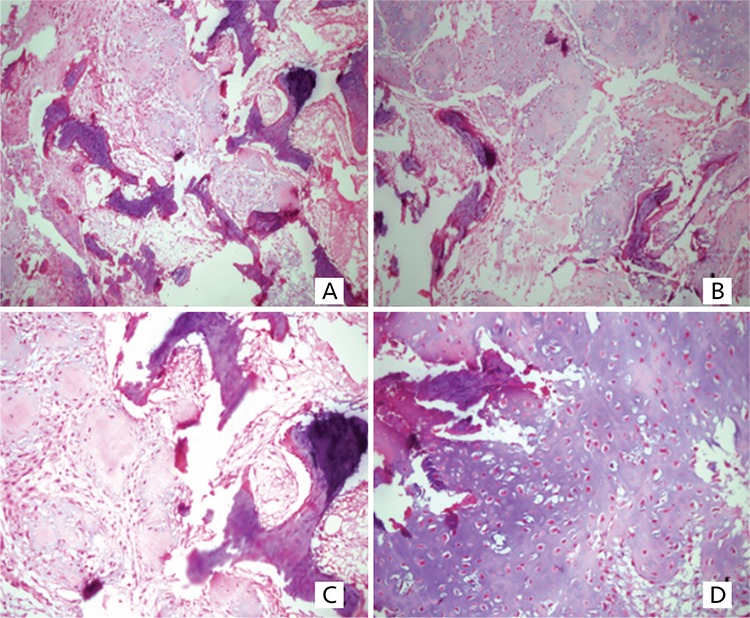
**A, B, C, D)** Tissue preparations of the nasal cavity-osteochondromyxoma: The tumor consists of lobular areas, with chondroid and predominantly osteoid cells. Focal mesencymal spindled cells are present (hematoxylin and eosin stain; magnification x200)

One year after the operation, at eight years of age, he presented with pubic hair. His physical examination revealed increased testicular volume. Central precocious puberty (CPP) was diagnosed with increased luteinizing hormone (LH) concentration and deranged LH to follicle stimulating hormone ratio on the gonadotropin releasing hormone (GnRH) test. GNRH analogue treatment was started. Cranial and pituitary MRI imaging was normal. Scrotal ultrasound (USG) revealed multiple bilateral macrocalcifications in the testes. At the age of nine years, he presented with difficulty in nasal breathing which was due to the recurrence of the intranasal tumor. Paranasal sinus computed tomography (CT) imaging revealed a lobulated, 22x25x28 mm mass with amorphous calcification, adjacent to the right frontal lobe, which extended to the base of the sphenoid sinus and which had destroyed surrounding tissues. Pituitary MRI was compatible with partial empty sella. At that point, the patient was referred to our clinic for endocrine evaluation and then followed up in conjunction with pediatric oncology and the otorhinolaryngology department. He was the first child of nonconsanguineous parents. His birth history was unremarkable and family history was not significant for tumor occurrence. On physical examination, his height was 146 cm [1.93 standard deviation (SD)], weight was 38.5 kg [1.25 SD] and body mass index was 18 kg/m^2^ (0.23 SD). On his right lateral lumbar area, there were two domed, soft papules, the largest diameter of which was about 5 mm (Figure 1). Bilateral testes volumes were 6 cc and the stretched length of the penis was 7cm. General examination was otherwise normal. Endocrine evaluation showed adrenocorticotropic hormone (ACTH) deficiency, peak cortisol level was 13.4 µg/dL during a low dose ACTH stimulation test. All other pituitary hormone concentrations were normal, except gonadotropins which were prepubertal due to GnRH analogue treatment. Hydrocortisone replacement was started and the patient was operated for recurrent intranasal mass. Mesenchymal tumor containing chondroid component was reported by histopathology (Figure 2). Due to infiltration of the adjacent bone by the tumor (Figure 2A, 2B, 2D), focal osteoid-like matrix within the tumor (Figure 2C), recurrence of the tumor and the radiological findings, a diagnosis of osteosarcoma could not be excluded. Nevertheless, clinical findings during the follow-up were not compatible with osteosarcoma and it was decided to follow the patient closely without starting chemotherapy. The testes were re-evaluated with scrotal ultrasonography at around the same time. Numerous coarse parenchymal calcifications in both testes and a 6x5 mm hypoechoic, heterogeneous, solid lesion in the left testis were detected. Two months after the operation, cranial MRI revealed a 31x13 mm residual mass. Therefore, positron emission tomography/CT (PET/CT) scan was used to evaluate the malignancy potential of the lesion. ^18^F-FDG PET imaging revealed normal uptake value in the tumor and also in other parts of the body.

**Figure 3:**
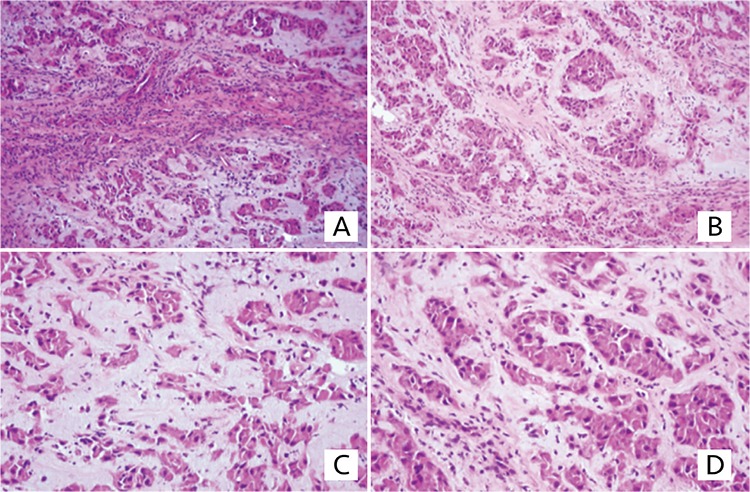
**A, B, C, D)** Tissue preparations of the testicular biopsy with large cell calcifying Sertoli cell tumor. The neoplastic cells form solid and hollow tubules and are immersed in a loose, myxoid matrix. The tumor is composed of large polygonal cells with abundant eosinophilic cytoplasm and eccentric nuclei (hematoxylin and eosin stain; magnification x100-200)

The patient was reoperated for the tumoral lesion in the nasal cavity and testicular biopsy was taken from the testicular lesions, considering that these lesions might be due to metastases of the primary disease. Histopathological examination of the intranasal lesion reported only granulation tissue and reactive bone formation were without tumoral tissue. However, testicular lesions were compatible with LCCSCT without malignant features (necrosis and mitosis) (Figure 3). The diagnosis of LCCSCT suggested the possibility of CNC. Robust histopathological diagnosis of the bone lesion was not clear, thus the specimens were re-evaluated for the possibility of OMX, which is known to be a rare CNC-associated bone tumor, most frequently located in the nasal sinuses and diaphyses of the long bones. Pathologic re-examination revealed osteoid and chondroid predominant lobular areas and focal mesenchymal spindle cells that suggested OMX. The patient was re-evaluated for fulfillment of the diagnostic criteria of CNC. The lesions on his back were cutaneous myxomas which are common components of CNC. Presence of OMX and LCCSCT, which are two major criteria, confirmed the diagnosis of CNC. Thus the patient was evaluated for other CNC-associated endocrinopathies, and growth hormone (GH) excess was detected. Pituitary MRI did not show any adenoma. Despite GH excess, growth velocity was not increased and other clinical manifestations related to GH excess were not found. No other endocrine dysfunction associated with CNC, such as hyperprolactinemia and hypercortisolemia, was detected. On the contrary, he had ACTH deficiency. For molecular confirmation, genetic analysis was performed and a heterozygous nonsense mutation, c.672G>A (p.Trp224*), was detected in the *PRKAR1A* gene. In this case, the malignancy potential of the LCCSCT was judged to be low by the histopathologist and the maximum diameter of the tumoral lesions was 6.7 mm. Therefore, the lesions were followed up with scrotal USG without intervention and the size of the tumoral lesions did not change during the subsequent 18 months follow-up. The patient was also screened for other CNC–associated tumors and no additional tumor was found.

## Discussion

CNC is a rare, multiple endocrine neoplasia syndrome with autosomal dominant inheritance. It is usually characterized by pigmented lesions of the skin and mucosal, cardiac, cutaneous and other myxomas, and multiple endocrine and non-endocrine tumors. To confirm the diagnosis, patients must meet at least two major criteria or one major and one supplemental criteria (5). In our case, CNC was diagnosed clinically with the coexistence of histologically proven OMX and LCCSCT as two major criteria and confirmed by genetic analysis. More than 80% of CNC patients develop spotty skin pigmentations, which typically appear early in life and may be located anywhere on the body, typically on the face, lips, genital area and mucosa (6). Pigmented skin lesions of CNC were not present in our case. Only two myxomatous lesions were detected as cutaneous manifestations.

CNC is characterized by endocrine overactivity. Primary pigmented nodular adrenocortical disease is the most common endocrine lesion and causes hypercortisolism (7). The adrenal imaging in this case were normal and he had hypocortisolism due to ACTH deficiency, which was associated with partial empty sella. Asymptomatic GH hypersecretion occurs in approximately 2/3 of patients, in most cases without imaging evidence of pituitary adenoma (5), as occurred in our patient.

In the case presented here the first manifestation of CNC was OMX, which is an extremely rare myxomatous tumor of bone, affecting 1% of CNC patients (8), but it is one of the 11 diagnostic criteria (5). Although he had presented with a bone tumor at the age of six, diagnosis of OMX was delayed by three years, because of the rarity of the tumor and the possibility of osteosarcoma. Awareness of OMX is poor due to the rarity of the tumor although diagnosis may be aided if there is a clinical suspicion of CNC of which the histopathologist is aware. Characteristically, OMX is a painless mass and may be unnoticed unless it enlarges and surrounds or invades vital structures. It may affect any bone, but is most frequently seen in the nasal sinuses and long bones of extremities (8). In our patient, the characteristic location of the tumor in the nasal sinus suggested OMX and it was subsequently proven histopathologically. Although OMX is a benign neoplasm, it can exhibit locally invasive characteristics (9). On imaging, OMX is well circumscribed, but can be destructive (8), as in our case. Osteosarcoma was considered in the differential diagnosis, because of the local invasive and destructive nature of the tumor and radiological findings. However, osteosarcoma was an unlikely diagnosis since no metastasis was observed during three years of follow-up. OMX has a good prognosis with complete excision, however local recurrence is very common with incomplete resection (8,9,10). Disease recurrence is therefore more likely at sites where complete resection is difficult, as in this case.

LCCSCTs, especially in children, present in association with CNC or Peutz-Jeghers syndrome (11). More than 75% of males with CNC may have LCCSCT (12). Malignancy is found in approximately 17% of patients, but is rare in young patients with bilateral tumors or in association with a genetic syndrome (11,13). Malignant behaviour is associated with large size (>4 cm), necrosis, increased mitotic activity, atypia and vascular invasion (14,15). In our case, bilateral multiple nodular lesions were present. The largest diameter of nodules was 6.7 mm. There was no evidence of malignancy histopathologically. Tumors may lead to premature epiphyseal fusion and induction of CPP, due to increased aromatase activity (5,14). Our case also presented with CPP. At first, it was thought that CPP was related to excision of the intranasal tumor close to the sellar region, but then it was realized that CPP may be associated with LCCSCT. In light of the low malignancy risk of this tumor, only imaging surveillance was performed, as recommended (14).

In conclusion, a case of CNC is reported, presenting with undiagnosed OMX as the first manifestation. OMX is a very rare tumor, but should be considered in the differential diagnosis of local invasive, recurrent intranasal tumors. In addition the possibility of LCCSCT should be kept in mind in cases of calcified testis tumor presenting with CPP.
